# Psychological Stress Risk Factors, Concerns and Mental Health Support Among Health Care Workers in Vietnam During the Coronavirus Disease 2019 (COVID-19) Outbreak

**DOI:** 10.3389/fpubh.2021.628341

**Published:** 2021-03-19

**Authors:** Phuong Thi Lan Nguyen, Tien Bao Le Nguyen, Anh Gia Pham, Khanh Ngoc Cong Duong, Mac Ardy Junio Gloria, Thanh Van Vo, Bay Van Vo, Toi Lam Phung

**Affiliations:** ^1^Social Economics and Administrative Pharmacy Program, Department of Pharmacy, Faculty of Pharmacy, Mahidol University, Bangkok, Thailand; ^2^Institute of Orthopaedics and Trauma Surgery, Viet Duc Hospital, Hanoi, Vietnam; ^3^Oncology Department, Viet Duc Hospital, Hanoi, Vietnam; ^4^Mahidol University Health Technology Assessment Graduate Program, Mahidol University, Bangkok, Thailand; ^5^School of Medicine, Vietnam National University, Ho Chi Minh City, Vietnam; ^6^Vietnam National University, Ho Chi Minh City, Vietnam; ^7^Department of Pharmacy, College of Pharmacy, University of the Philippines Manila, Manila, The Philippines; ^8^Department of Surgery, Hanoi Medical University, Hanoi, Vietnam; ^9^Department of Pharmacy, Thong Nhat Hospital, Ho Chi Minh City, Vietnam; ^10^Health Strategy and Policy Institute, Ministry of Health, Hanoi, Vietnam

**Keywords:** COVID-19, healthcare worker, risk factor, psychological stress, mental health

## Abstract

**Introduction:** Coronavirus disease 2019 (COVID-19) has significantly affected health care workers (HCWs), including their mental health. However, there has been limited evidence on this topic in the Vietnamese context. Therefore, this study aimed to explore COVID-19-related, psychological stress risk factors among HCWs, their concerns and demands for mental health support during the pandemic period.

**Methods:** We employed a cross-sectional study design with convenience sampling. An online, self-administered questionnaire was used and distributed through social media among medical and non-medical HCWs from April 22 to May 12, 2020. HCWs were categorized either as frontline or non-frontline. We measured the prevalence of psychological stress using the Impact of Event Scale-Revised (IES-R) instrument. Multivariate binary logistic regression analysis was performed to identify risk factors associated with psychological stress among HCWs.

**Results:** Among the 774 enrolled participants, 761 (98.3%) eligible subjects were included in the analysis. Most respondents were females (58.2%), between 31 and 40 years of age (37.1%), lived in areas where confirmed COVID-19 cases had been reported (61.9%), medical HCWs (59.9%) and practiced being at the frontline (46.3%). The prevalence of stress was 34.3%. We identified significant risk factors such as being frontline HCWs (odds ratio [OR] = 1.77 [95% confidence interval [CI]: 1.17–2.67]), perceiving worse well-being as compared to those before the COVID-19 outbreak [OR = 4.06 (95% CI: 2.15–7.67)], and experiencing chronic diseases [OR = 1.67 (95% CI: (1.01–2.77)]. Majority (73.9%) were concerned about testing positive for COVID-19 and exposing the infection to their families. Web-based psychological interventions that could provide knowledge on managing mental distress and consulting services were highly demanded among HCWs.

**Conclusion:** The prevalence of psychological stress among HCWs in Vietnam during the COVID-19 pandemic was high. There were also significant risk factors associated with it. Psychological interventions involving web-based consulting services are highly recommended to provide mental health support among HCWs.

## Introduction

At the end of 2019, the world witnessed the emergence of a novel pneumonia caused by a severe acute respiratory syndrome coronavirus 2–SARS-CoV-2, known as the coronavirus disease 2019 (COVID-19) (1). COVID-19 was then declared a global pandemic by the World Health Organization (WHO) on 11 March 2020 with 118,000 confirmed cases and 4,291 deaths across 114 countries (2). Its rapid surge from thousands up to millions of cases has raised attention worldwide (3). COVID-19 hit Vietnam for the first time on 23 January 2020. Also, last 20 March 2020, Vietnam documented the first two COVID-19 cases in female nurses at a frontline hospital in the country's capital (4). The country initially succeeded in fighting the first wave of the disease and its transmission since 22 April 2020. (4). However, on 25 July 2020, a positive case which was identified at a provincial hospital marked the resurgence of the outbreak (4). On 13 September 2020, Vietnam had documented 1,060 cases with 910 recoveries and 35 deaths (4).

During the pandemic, Vietnamese government has implemented preventive measures such as the nationwide closure of schools and non-essential businesses, border control, social distancing, and regulation for wearing masks in public in order to contain the spread of COVID-19 (5, 6). Furthermore, healthcare workers (HCWs) have played a vital role in battling against COVID-19, not only for providing care to the society, but also directly being involved in the treatment of confirmed cases among hospitals designated for COVID-19 care in the country (7). HCWs contributed to numerous epidemiological actions, including early detection of cases, isolation, tracing cases, and close monitoring of either suspected cases or close contact groups (6, 7). While the general public had been urged to maintain social distancing and remain at home to minimize disease transmission, HCWs were prepared to do the opposite (5). As such, HCWs are more likely to have psychological health problems such as stress, anxiety, depression and insomnia (8).

The negative psychological burden of working under an infectious disease environment is prominent and inevitable (9).

Previous evidence suggested that HCWs were emotionally affected and traumatized during outbreaks, like in the case of severe acute respiratory syndrome (SARS) in 2003 (10, 11). In fact, HCWs during an outbreak might experience the fear of being infected and other unfavorable conditions, such as increasing number of confirmed cases, excessive workload, shortage of personal protective equipment, and intense media scrutiny, that could increase their risk of developing psychological problems (12). They, therefore, suffered from sleep disorders with worse sleep quality and sleep time reduction aside from anxiety and guilt (13). An observational cohort study in the United Kingdom and the United States of America indicated that frontline HCWs were 11 times more likely to contract COVID-19 than the general community (14). Such psychological impact would not only burden HCWs' well-being but might also hinder their ability to effectively manage COVID-19 (15, 16). Furthermore, a systematic review on the mental health problems faced by HCWs during the COVID-19 pandemic reported that the disease can be an independent risk factor for stress among HCWs (17).

Vietnam documented the first two COVID-19 cases on HCWs which were nurses at a frontline hospital in the first wave of the pandemic. Alarmingly, the resurgence of the outbreak in Vietnam in July at a provincial hospital had added 14 COVID-19 cases among HCWs (4). As such, Vietnam's Ministry of Health has made efforts to alleviate the pressure for HCWs, such as sending more medical staff to reduce workload, implementing infection control programs, giving personal protective equipment, and providing financial support to frontline HCWs (4). Nevertheless, these efforts focused only on managing their physical health. With the unpredictability and complexity of COVID-19, understanding its psychological impacts for the timely provision of mental health support to HCWs is essential during the pandemic. Notably, there has been limited evidence on the psychological impact among HCWs, their concerns, as well as the mental health support demand during the COVID-19 pandemic in Vietnam. Therefore, this present study aimed to examine the psychological stress among HCWs, identify factors associated with it, and explore the demand for psychological support among HCWs during COVID-19 pandemic.

## Materials and Methods

### Study Population

A cross-sectional study design was conducted using a self-administered questionnaire. HCWs were defined as people engaged in providing and delivering care and services to patients (18). The inclusion criteria are subjects being a HCW, working in the health facilities in Vietnam, volunteering to participate in the study. The study excluded people working in other sectors not related to health care, people working in health facilities but not involving in delivering care and services to patients such as accountant, information technology, recruiter, and security. Data were collected from 24 April to 12 May 2020.

Frontline HCWs were people involved in the epidemiological investigation, testing, treatment and management of COVID-19 patients, while non-frontline HCWs included healthcare staff who were providing services to patients not related to COVID-19 (19). Physicians and nurses were categorized as medical HCWs, while pharmacists and health technicians were classified as non-medical HCWs (20).

### Questionnaire

A survey questionnaire was developed to obtain data for research. This included information on socio-demographic characteristics, and a tool to assess HWCs' mental health, their concerns related to COVID-19 and their need for mental health support.

### Socio-Demographic Characteristics

This part included age, sex (male or female), living area (having COVID-19 cases or not), marital status (single, married or divorced/separated/widowed), educational attainment (intermediate, college, undergraduate or post-graduate), job category (medical HCW or non-medical HCW), working department (treatment or prevention), health facility level (central, provincial, district, commune or private health facility), years of working experience (<5, 5–10, 10–20 or >20 years), whether being at the frontline in the COVID-19 taskforce, whether the respondent had chronic diseases, self-perceived change in respondent's health status (better, almost unchanged, worse or much worse), whether the respondent experienced being quarantined, change in workload during COVID-19 outbreak (increased, unchanged, decreased or temporary off). In Vietnamese context, people with “intermediate degree” in educational attainment refers to those who entered medical high school. Frontline HCWs were defined as those directly engaged in treating patients with confirmed COVID-19.

### Mental Health Assessment

The Impact of Event Scale-Revised (IES-R) was used to assess the psychological response associated with trauma for different specific life events (21). Previous studies reported that this questionnaire could assess stress conditions with satisfactory reliability and validity among Vietnamese and with other countries' sample population (22–26). The scale consisted of three dimensions (avoidance, intrusion, and hyperarousal) with a total of 22 entries. Responses were based on a five-point Likert scale (not at all, a little bit, moderately, quite a bit and extremely). The total score ranged from 0 to 88, with a higher score corresponding to a greater stress level. The result was interpreted based on four groups: normal (score 0–23), mild (score 24–32), moderate (score 33–36), and severe (score ≥ 37) stress.

### COVID-19 Concerns and Mental Health Support

To determine COVID-19-related concerns among HCWs and their need for mental health support, questions were adapted from previous studies (27, 28) and modified to reflect Vietnamese context. In the paper about understanding HCWs during the COVID-19 pandemic, they indicated eight sources of anxiety among HCWs when working under the pandemic (28). We utilized these sources to ask whether Vietnamese HCWs concerned about it. Regarding mental health support, three multiple-choice questions were asked to explore the content that HCWs were interested in, their preferred resources, and who they would like to receive care from. These contents were adapted from Kang's study which explored the psychological care need among medical and nursing staff in Wuhan (27).

### Data Collection

An online questionnaire was established using the Google form platform due to the social distancing strategy and restriction of face-to-face contact. Using convenience sampling, the survey link was circulated through personal contacts and social media networks (e.g., Facebook) and mobile apps in Vietnam (e.g., Zalo). The respondents accessed the link and voluntarily responded to the survey.

### Ethical Consideration

The study protocol was approved by the Institutional Review Board of Thong Nhat Hospital, Ho Chi Minh city, Vietnam (IRB approval number: 10/BB-BVTN). The questionnaires used in the study were anonymized. Informed consent of the participants was obtained prior to data collection.

### Data Analysis

We performed descriptive statistics and presented the results as frequency (percentage) or median with an interquartile range (IQR). The score of the IES-R was expressed as median (IQR). Chi-square test or Fisher's exact test was employed to examine the associations among stress, COVID-19-related concerns and needs of mental health support, where appropriate. Univariate and multivariable logistic regression analyses were conducted to identify risk factors associated with psychological stress. The IES-R scores of <23 and ≥23 were classified as normal psychological status and having psychological stress, respectively (21). Respondents' socio-demographic characteristics were considered as explanatory factors. A *p*-value of < 0.25 in the univariate analysis was used as a cut-off point to include variables in multivariable logistic regression (29). The strength of associations among variables was reported as odds ratios (OR) with 95% confidence interval (CI). A *p*-value ≤ 0.05 was considered statistically significant. All analyses were performed using Stata version 14.0 (StataCorp, College Station, Texas 77845 USA).

## Results

### Sociodemographic Characteristics

From a total of 774 HCWs who completed the survey, 761 (98.3%) subjects were eligible for analysis. Table 1 shows the socio-demographic characteristics of the respondents. We found that the median age was 34 years, where the 31–40 age group accounted for the highest proportion (37.1%). Most were females (58.2%), lived in areas with confirmed COVID-19 cases (61.9%), married (64.5%), attained undergraduate level (51.9%), and medical HCWs (59.9%). Also, many respondents worked in a treatment unit (78.1%), at a provincial health facility (33.5%) and had 5–10 years of working experience (30.2%). A total of 211 (27.7%) respondents were frontline HCWs in the COVID-19 taskforce. Also, about 12.0% had chronic diseases and 14.1% experienced quarantine. When asked regarding their current health status as compared with that before COVID-19 outbreak and their workload during the COVID-19 outbreak, majority (85.8%) answered as unchanged.

**Table 1:** Socio-demographic characteristics among HCWs during the COVID-19 outbreak in Vietnam (*n* = 761).

**Variables**	***n* (%)**
**Age group**	
18–25	77 (10.1)
26–30	193 (25.4)
31–40	282 (37.1)
>40	209 (27.4)
**Gender**	
Male	318 (41.8)
Female	443 (58.2)
**Living area having COVID-19 cases**	
Yes	471 (61.9)
No	290 (38.1)
**Marital status**	
Single	247 (32.5)
Married	491 (64.5)
Divorced, separated, or widowed	23 (3.0)
**Educational attainment**	
Intermediate	67 (8.8)
College	72 (9.5)
Undergraduate	395 (51.9)
Postgraduate	227 (29.8)
**Job title**	
Medical HCW	456 (59.9)
Non-medical HCW	305 (40.1)
**Working Department**	
Treatment	594 (78.1)
Preventive health	97 (12.7)
Community care	32 (4.2)
Others^a^	38 (5.0)
**Experience in being quarantined**	
Yes	107 (14.1)
No	654 (85.9)
**Health facility levels**	
Central health facility	194 (25.5)
Provincial health facility	255 (33.5)
District health facility	121 (15.9)
Commune health station	19 (2.5)
Private health facility	151 (19.8)
Others^a^	21 (2.8)
**Working experience (years)**	
<5	206 (27.1)
5–10	230 (30.2)
10–20	207 (27.2)
>20	118 (15.5)
**Responsibility for COVID-19 taskforce**	
Frontline	211 (27.7)
Non-frontline	550 (72.3)
**Chronic disease**	
Yes	91 (12.0)
No	670 (88.0)
**Self-perceived health status compared to before COVID-19 outbreak**	
Better	48 (6.3)
Almost unchanged	653 (85.8)
Worse	60 (7.9)
Much worse	0 (0.0)
**Workload during COVID-19 outbreak**	
Increased workload	88 (11.6)
Unchanged workload	413 (54.3)
Decreased workload	220 (28.9)
Temporary off work	40 (5.2)

a *Schools, universities, research institutes, pharmaceutical companies*.

### Stress Severity Level and Predictors of the Psychological Stress Outcome

We found that 34.3% HCWs had psychological stress symptoms. Supplementary Tables 1, 2 (Supplementary Data) illustrate the results from the logistic regression models of potential predictors of psychological stress among HCWs. From these, we identified significant risk factors, such as being frontline HCWs [OR = 1.77 (95% CI: 1.17–2.67)], perceiving as worse health status as compared to that before the COVID-19 outbreak [OR = 4.06 (95% CI: 2.15–7.67)], and having chronic diseases [OR = 1.67 (95% CI: (1.01–2.77)]. As for educational attainment, HCWs having either a college degree or a bachelor's degree showed less stress as compared with those who got an intermediate degree [OR = 0.30 (95% CI: 0.14–0.69), OR = 0.45 (95% CI: 0.25–0.81), respectively]. As for marital status, HCWs who were divorced, separated, or widowed were less likely to have psychological stress as compared with those who were single [OR = 0.18 (0.04–0.86)].

### COVID-19-Related Concerns

Table 2 presents the COVID-19-related concerns among HCWs. When asked about their concerns related to working during the outbreak, majority (73.9%) answered being exposed to COVID-19 at work and taking the infection to their families. This was followed by a concern on access to appropriate personal protective equipment (56.5%). Some participants (37.3%) were also worried about not having a rapid access to testing if they developed COVID-19 symptoms and the concomitant fear of propagating infection at work. Only 10.2% felt nervous about having limited access to up-to-date information and communication. As for stress severity level, HCWs were the most anxious about being exposed to COVID-19 at work and taking the infection home (>70% in all levels). Notably, the proportion of respondents having COVID-19-related concerns increased with higher stress level.

**Table 2:** The association between stress severity level of HCWs and their COVID-19-related concerns during the COVID-19 outbreak in Vietnam.

**Concerns**	**Severity of stress [Number (%)]**					***p*-value**
	**Total (*n* = 761)**	**Normal (*n* = 500)**	**Mild (*n* = 113)**	**Moderate (*n* = 51)**	**Severe (*n* = 97)**	
Access to appropriate personal protective equipment	430 (56.5)	271 (54.2)	61 (54.0)	29 (56.9)	69 (71.1)	0.020
Being exposed to COVID-19 at work and taking the infection home to their family	562 (73.9)	351 (70.2)	92 (81.4)	45 (88.2)	74 (76.1)	0.006
Not having rapid access to testing if they develop COVID-19 symptoms and concomitant fear of propagating infection at work	284 (37.3)	167 (33.4)	44 (39.0)	28 (54.9)	45 (46.4)	0.004
Uncertainty that their organization will support/take care of their personal and family needs if they develop infection	237 (31.1)	132 (26.4)	39 (34.5)	22 (43.1)	44 (45.4)	<0.001
Access to childcare during increased work hours and school closures	248 (32.6)	129 (25.8)	54 (47.8)	22 (43.1)	43 (44.3)	<0.001
Support for other personal and family needs as work hours and demands increase (food, hydration, lodging, transportation)	186 (24.4)	89 (17.8)	37 (32.7)	20 (39.2)	40 (41.2)	<0.001
Being able to provide competent medical care if deployed to a new area (e.g., non-ICU nurses having to function as ICU nurses)	224 (29.4)	131 (26.2)	38 (33.6)	15 (29.4)	40 (41.2)	0.019
Lack of access to up-to-date information and communication	78 (10.2)	40 (8.0)	11 (9.7)	10 (19.6)	17 (17.5)	0.004

### Psychological Care Needs

Table 3 shows the results on the psychological care needs among HCWs. As for contents of interest, among HCWs with normal psychological status, almost half (43.8%) did not feel the necessity to have access to mental health support. Nevertheless, those with either severe or moderate stress levels wanted to gain skills to self-rescue themselves and to help others alleviate psychological distress. As for the desired platform to obtain such competence, half of the respondents preferred a website where they can access psychological knowledge. When they were asked about whom they would like to receive care from, most answered friend or colleagues (37.2%) or psychologists and psychiatrists (31.9%). Among HCWs with severe stress levels, most (48.5%) agreed to need help from psychologists.

**Table 3:** The association between stress severity level and needs of mental health support among HCWs during the COVID-19 outbreak in Vietnam.

**Content of interest**	**Severity of stress [Number (%)]**					*p*-value*
	**Total (*n* = 761)**	**Normal (*n* = 500)**	**Mild (*n* = 113)**	**Moderate (*n* = 51)**	**Severe (*n* = 97)**	
Knowledge of psychology	264 (34.7)	163 (32.6)	47 (41.6)	18 (35.3)	36 (37.1)	0.308
Skills for self-rescue	284 (37.3)	154 (30.8)	52 (46.0)	25 (49.0)	53 (54.6)	** <0.001**
Skills for help others alleviate psychological distress	258 (33.9)	136 (27.2)	42 (37.2)	30 (58.8)	50 (51.6)	** <0.001**
Seek help from psychologists or psychiatrists	78 (10.2)	35 (7.0)	13 (11.5)	8 (15.7)	22 (22.7)	** <0.001**
Not necessary	292 (38.4)	219 (43.8)	39 (34.5)	12 (23.5)	22 (22.7)	** <0.001**
**Resources**						
Paper sources (books, brochures, etc.)	127 (16.7)	63 (12.6)	18 (15.9)	21 (41.2)	25 (25.8)	** <0.001**
Website provided with psychological resources	382 (50.2)	241 (48.2)	52 (46.0)	29 (56.9)	60 (61.9)	0.051
Group psychotherapy	74 (9.7)	42 (8.4)	7 (6.2)	7 (13.7)	18 (18.6)	**0.007**
Individual counseling and psychotherapy	74 (9.7)	38 (7.6)	11 (9.7)	8 (15.7)	17 (17.5)	**0.010**
Uninterested	7 (0.9)	6 (1.2)	0 (0.0)	0 (0.0)	1 (1.0)	0.579**
**Prefer to receive care from**						
Psychologists or psychiatrists	258 (33.9)	153 (30.6)	42 (37.2)	16 (31.4)	47 (48.5)	**0.006**
Family or relatives	243 (31.9)	147 (29.4)	41 (36.3)	19 (37.3)	36 (37.1)	0.229
Friends or colleagues	283 (37.2)	160 (32.0)	45 (39.8)	29 (56.9)	49 (50.5)	** <0.001**
Do not need help	25 (3.3)	16 (3.2)	6 (5.3)	0 (0.0)	3 (3.1)	0.399**

*Chi-square test/**Fisher's exact test. Bold values have a meaning that they are less than 0.05 which indicated that there was a significant difference between severity of stress and the need for those content of interest.

## Discussion

To the authors' knowledge, this study is the first mental health investigation amongst HCWs in the wake of COVID-19 outbreak in Vietnam. The analysis included 761 respondents, with a 34.3% prevalence of psychological stress symptoms among them. The study showed that majority were females, aged 31–40 years, lived in areas having confirmed COVID-19 cases, medical HCWs, attained undergraduate level, married, and worked in provincial health facilities at the treatment unit for 5–10 years. We also found that working in the COVID-19 task force team, perceiving a worse well-being compared to that before the COVID-19 outbreak, having chronic diseases were independent predictors for having psychological stress outcomes. Most HCWs were concerned about their fear of being exposed to COVID-19 and taking the infection home. Findings also suggested the demand for psychological support, in which, most of HCWs wished to have a website provided with psychological knowledge. Moreover, those who have severe stress levels would like to receive care from either psychiatrists or their colleagues.

In this study, a proportion of 34.3% of HCWs reported stress symptoms. This finding was similar to the stress prevalence related to COVID-19 among the Vietnamese general population (35.9%) (30). Compare to the pooled prevalence of stress in Asia, this figure was a little bit lower (34.3% vs. 41.3%) (31). Notably, this figure was about half of the prevalence in China as reported in a study conducted by Lai et al. where more than 70.0% had stress disorders during the outbreak (19). This could be explained by the fact that the outbreak's impact in China, which was the original epicenter of the coronavirus, was more serious and acute as compared with Vietnam. Particularly, during the time when these two studies were conducted, Vietnam had only 413 COVID-19 cases, while China had more than 80,000 positive cases (3). Another reason was that our sample population focused on HCWs from all sectors, while Lai et al. employed a hospital-based survey, which may involve higher risks for exposure to COVID-19 and thus could lead to increased fear of spreading the virus and being isolated (19). The prevalence was also much lower compared to that in USA of 60.2% (31). Although USA is a high-income country with advanced health care system, HCWs in this country still suffered from stress because of the overwhelming COVID-19 cases and deaths.

Notably, HCWs working at the frontline had at least 2-fold increased risk for having psychological stress as compared with those not at the frontline. This finding resonated with Lai's study in China and Alshekaili's study in Oman which reported that frontline HCWs were around 1.5 times more likely to develop psychological stress than non-frontline HCWs (19, 32). Evidently, having direct and frequent contact with confirmed or suspected COVID-19 patients has been well-recognized to render frontline HCWs vulnerable to suffer from stress disturbances as it places them at an increased risk of infection that might threaten their lives (14). In Italy, as of 16 April 2020, about 17,000 frontline HCWs tested positive to COVID-19, and 127 of them succumbed to the disease (33). This implies that HCWs are under a huge risk of threatening their lives due to the COVID-19 crisis, which in turn could lead to higher psychological stress. As such, our study suggests that continuous efforts aimed at improving the mental well-being should be focused among HCWs who are directly treating and managing patients with COVID-19. Moreover, we found that HCWs who perceived a negative change in their health status as compared with that before COVID-19 were likely to have psychological stress. This finding conformed with another cross-sectional study on mental health among medical and nurse staff where the group perceiving their health status as worse than before COVID-19 had the highest prevalence of severe stress (27).

Furthermore, we also found evidence that the risk of having stress among HCWs with chronic diseases was double that of those without such diseases. Indeed, chronic diseases, such as heart disease, stroke, diabetes mellitus, and cancer may increase susceptibility to COVID-19 (34). This was proven in a meta-analysis where patients with pre-existing chronic diseases were about 3.5 times more likely to develop severe COVID-19 and be admitted to an intensive care unit as compared with those who had none (34). Specifically, compared to COVID-19 patients with no pre-existing chronic diseases, COVID-19 patients who had diabetes, hypertension, cardiovascular disease, or chronic pulmonary disease had a higher risk of developing severe disease, with an OR (95% CI) of 2.61 (1.93–3.52), 2.84 (2.22–3.63), 4.18 (2.87–6.09), and 3.83 (2.15–6.80), respectively (34). Therefore, future intervention should be targeted to these groups.

HCWs who attained a college degree or bachelor's degree were less likely to have psychological stress than those with intermediate degree. This may be explained by the fact that higher educational background may have more professional knowledge on exposure patterns and transmission characteristics of COVID-19 (35–37); thus, they might have a good awareness and understanding of the disease and could manage better their situations. Likewise, divorced HCWs were less likely to have psychological stress than those who were single. This difference may be due to the maturity between these subjects, such that those who failed in their marriage may have gone through more challenges and events as they got older which helped them to deal with the crisis better.

As for concerns when working during the pandemic, most HCWs expressed their hope of not acquiring the infection so that they can protect their family from being infected since they may be a potential carrier for COVID-19 transmission. Despite of the adequate provision of personal protective equipment (PPE) for HCWs in Vietnam, this concern was unavoidable because COVID-19 exposure among Vietnamese HCWs were high. Indeed, in Vietnam, HCWs contributed on all fronts from prevention, screening to diagnosis and treatment of COVID-19 when the COVID-19 hit the country (6). Moreover, the Vietnamese government imposed extensively an intensive tracing and tracking of people who have been contacted with infected people; when a confirmed case was identified, the authorities would trace contact from the confirmed case to the fourth level of contact (6). This required a large number of HCWs workforce involving in COVID-19 testing for a high volume of patients with different degrees of pathology and severity. Noteworthy, their concern was exacerbated by the risk of working place, the increased workload and information from social media in the country as well (6, 38). High risk of infection of HCWs was also related to the pernicious and unpredictable characteristic of COVID-19 as asymptomatic infections (39). Furthermore, proper caution is advised considering that a vaccine to address the outbreak is still being developed (40).

Apart from social demographic factors discussed in the study context, other factors were found to be related stress outcomes among HCWs. Banerjee reported that in China and the United Kingdom (UK) which are countries with high COVID-19 cases, physician developed stress due to the increased witness to death, the increased risk of exposure and self-blame, and guilt of spreading the infection to the family members (13). Psychiatric disorders such as anxiety, sleep disturbances, and depression commonly co-occurs with posttraumatic stress disorder (41). In the systematic review of psychological well-being in South Asia, these psychological comorbidities were reported to be suffered by HCWs in two studies in India (13).

As for the needs for mental health support, HCWs with severe or moderate stress levels wanted to have skills for managing themselves and for helping others relieve psychological distress. They also preferred a website to access such knowledge. Friends or colleagues were the ones HCWs trusted the most to share their problems. Since this was the first study in the country that examined the concerns and demands regarding mental health support among HCWs, our results suggest that interventions should focus on developing a web-based platform that could provide psychological resources which might help HCWs perceive and manage stress. Also, HCWs' friends and families should be encouraged to access this website so that they can help and support them to recognize, understand and overcome psychological issues. The interface and content of the website should be less complicated and easily comprehensible so that both HCWs and the general population could apply the knowledge in improving their mental health and assert their vital role when they have a HCW family member working during the pandemic. In addition, telehealth project has been released in Vietnam to provide remote consultation and treatment since September 2020 (42). Thereby, the authorities could take tele-psychological services into considerations during the pandemic. However, the psychological-mindedness of the HCWs should also be considered as whether they are willing to seek help or feel social stigma.

Some limitations should be highlighted in this study. Firstly, a self-administered questionnaire through online platform could be biased depending upon the respondents' mood prior to answering it. Whereas, they might feel too burdened to respond to the survey or not at all interested in answering it. Second, the study employed a cross-sectional design within 2 weeks and did not assess the psychological stress symptoms in the following period. Because of the negative unpredictable situation brought about by the COVID-19 outbreak, the respondents' psychological symptoms could become worse after the period of data collection. Third, lack of qualitative component to understand the concerns of HCWs in depth. Moreover, the study did not exclude people with a history childhood abuse or who are being treated with psychological problems, which might overestimate the study results. Lastly, this study used only a single scale (IES-R) which focused only the psychological stress aspect without other common psychiatric comorbidities. As such, consideration should be given to the extent other mental health problems, such as insomnia, depression, or anxiety, which could provide a more comprehensive picture of the mental health impact on HCWs during the outbreak. Given these limitations, further studies covering more psychological aspects are recommended and employing a probability sampling technique with a larger sample size would be needed to verify the results.

## Conclusion

Overall, this study highlighted the prevalence of psychological stress among HCWs during the COVID-19 period in Vietnam. It was found that there was a higher risk of having stress among HCWs who were at the frontline, perceived their health status as worse as compared with that before the pandemic, and experienced chronic diseases. Most HCWs were worried about being a potential source of infection to their families. Given the context of an ongoing resurgence of COVID-19 in the country and with more complicated cases whose some of their sources were still not known, the mental health of HCWs may deteriorate more and get worse. Therefore, psychological interventions are highly recommended to provide mental health support among HCWs. Additionally, health authorities should be responsible in protecting the psychological well-being of HCWs during the pandemic. Furthermore, further research will be needed to investigate comprehensively psychological well-being of HCWs during and post-pandemic along with policy strategies to measure it properly.

## Data Availability Statement

The original contributions generated for this study are included in the article/Supplementary Material, further inquiries can be directed to the corresponding author/s.

## Ethics Statement

The studies involving human participants were reviewed and approved by Institutional Review Board of Thong Nhat Hospital, Ho Chi Minh city, Vietnam. The patients/participants provided their written informed consent to participate in this study.

## Author Contributions

PN, TN, AP, KD, MG, TV, BV, and TP: conceptualization and writing—review and editing. PN, TN, AP, KD, BV, and TP: methodology. PN, TN, AP, KD, MG, TV, and TP: data curation. PN, TN, AP, KD, and MG: data analysis. PN, KD, TV, and AP: writing—original draft preparation. TP: supervision. PN, TN, AP, KD, and TP: project administration. All authors contributed to the article and agreed to the submitted version of the manuscript.

## Conflict of Interest

The authors declare that the research was conducted in the absence of any commercial or financial relationships that could be construed as a potential conflict of interest.
