# Supplementary material for: Psychological Stress Risk Factors, Concerns and Mental Health Support Among Health Care Workers in Vietnam During the Coronavirus Disease 2019 (COVID-19) Outbreak
Source: Front Public Health. 2021 Mar 19;9:628341. doi: 10.3389/fpubh.2021.628341 (PMC8017279; doi:10.3389/fpubh.2021.628341)
Supplement: Supplementary file 1 [file Data_Sheet_1.docx]

Supplementary data

Psychological stress risk factors, concerns and mental health support among health care workers in Vietnam during the coronavirus disease 2019 (COVID-19) outbreak

**Content**

**Supplemental Tables: Stress severity level and predictors of the psychological stress outcome**

Table S1. Univariate logistic regression analysis of risk factors for psychological stress using the IES-R among HCWs under the COVID-19 outbreak in Vietnam

Table S2. Multivariate logistic regression analysis of risk factors for psychological stress using the IES-R among HCWs under the COVID-19 outbreak in Vietnam

Table S1. Univariate logistic regression analysis of risk factors for psychological stress using the IES-R among HCWs under the COVID-19 outbreak in Vietnam

| **Variables** | **OR (95%CI)** | **P-value** | **Variables** | **OR (95%CI)** | **P-value** |
| --- | --- | --- | --- | --- | --- |
| **Age groups** |  |  | **Health facility levels** |  |  |
| 18-25 | 1 [Reference] | **0.016** | Central health facility | 1 [Reference] | **0.015** |
| 26-30 | 1.22 (0.68-2.22) |  | Provincial health facility | 0.83 (0.56-1.21) |  |
| 31-40 | 1.43 (0.81-2.51) |  | District health facility | 0.73 (0.46-1.18) |  |
| >40 | 2.11 (1.19-3.77) |  | Commune health center | 1.65 (0.64-4.25) |  |
| **Gender** |  |  | Private health facility | 0.50 (0.31-0.80) |  |
| Male | 1 [Reference] | 0.211 | Others^a^ | 0.35 (0.11-1.08) |  |
| Female | 1.21 (0.89-1.65) |  | **Working experience (years)** |  |  |
| **Living area having COVID-19 cases** |  |  | <5 | 1 [Reference] | 0.051 |
| No | 1 [Reference] | **0.015** | 5-10 | 0.69 (0.47-1.02) |  |
| Yes | 1.48 (1.08-2.02) |  | 11-20 | 0.65 (0.44-0.98) |  |
| **Marital status** |  |  | >20 | 0.54 (0.33-0.88) |  |
| Single | 1 [Reference] | **0.003** | **Responsibility for COVID-19 taskforce** | |  |
| Married | 1.34 (0.96-1.85) |  | Non-frontline | 1 [Reference] | **<0.001** |
| Divorced, separated, or widowed | 0.21 (0.05-0.94) |  | Frontline | 2.11 (1.53-2.93) |  |
| **Educational attainment** |  |  | **Chronic disease** |  |  |
| Intermediate | 1 [Reference] | **0.005** | No | 1 [Reference] | **<0.001** |
| College | 0.49 (0.24-1.02) |  | Yes | 2.16 (1.39-3.37) |  |
| Undergraduate | 0.65 (0.38-1.10) |  | **Self-perceived health status compared to before COVID-19 outbreak** | | |
| Postgraduate | 1.09 (0.62-1.89) |  | Almost unchanged | 1 [Reference] | **<0.001** |
| **Job title** |  |  | Better | 0.49 (0.23-1.02) |  |
| Non-medical HCW | 1 [Reference] | **<0.001** | Worse | 4.92 (2.77-8.76) |  |
| Medical HCW | 1.85 (1.35-2.54) |  | Much worse |  |  |
| **Working Department** |  |  | **Experience in being quarantined** | |  |
| Community care | 1 [Reference] | 0.107 | No | 1 [Reference] | 0.171 |
| Treatment | 1.98 (0.84-4.66) |  | Yes | 1.34 (0.88-2.04) |  |
| Preventive health | 1.93 (0.76-4.92) |  | **Workload during COVID-19 outbreak** | |  |
| Others^a^ | 0.95 (0.30-2.99) |  | Unchanged workload | 1 [Reference] | **0.002** |
|  |  |  | Increased workload | 2.28 (1.43-3.64) |  |
|  |  |  | Decreased workload | 1.35 (0.96-1.91) |  |
|  |  |  | Temporary off work | 0.66 (0.31-1.43) |  |
| Note: ^a:^ Schools, universities, research institutes, pharmaceutical companies | | | | | |

Table S2. Multivariate logistic regression analysis of risk factors for psychological stress using the IES-R among HCWs under the COVID-19 outbreak in Vietnam

| **Variables** | | **No. of cases (%)** | **OR (95%CI)** | **P-value** |
| --- | --- | --- | --- | --- |
| **Responsible for COVID-19 taskforce** | |  |  |  |
|  | Non-frontline | 162 (62.1) | 1 [Reference] | **-** |
|  | Frontline | 99 (37.9) | 1.77 (1.17-2.67) | **0.007** |
| **Self-perceived health status compared to before COVID-19 outbreak** | |  |  |  |
|  | Almost unchanged | 210 (80.5) | 1 [Reference] | **-** |
|  | Better | 9 (3.5) | 0.57 (0.26-1.26) | 0.164 |
|  | Worse | 42 (16.0) | 4.06 (2.15-7.67) | **<0.001** |
|  | Much worse | 0 (0.0) | - | - |
| **Chronic disease** | |  |  |  |
|  | No | 215 (82.4) | 1 [Reference] | **-** |
|  | Yes | 46 (17.6) | 1.67 (1.01-2.77) | **0.046** |
|  | **Job title** |  |  |  |
|  | Non-medical HCW | 181 (69.3) | 1 [Reference] | - |
|  | Medical HCW | 80 (30.7) | 1.16 (0.77-1.75) | 0.464 |
|  | **Workload during COVID-19 outbreak** |  |  |  |
|  | Unchanged workload | 126 (48.3) | 1 [Reference] | - |
|  | Increased workload | 44 (16.8) | 1.48 (0.82-2.68) | 0.190 |
|  | Decreased workload | 82 (31.4) | 1.13 (0.74-1.72) | 0.580 |
|  | Temporary off work | 9 (3.5) | 0.42 (0.17-1.04) | 0.061 |
|  | **Marital status** |  |  |  |
|  | Single | 76 (29.1) | 1 [Reference] |  |
|  | Married | 183 (70.1) | 0.93 (0.60-1.44) | 0.747 |
|  | Divorced, separated, or widowed | 2 (0.8) | 0.18 (0.04-0.86) | **0.031** |
|  | **Educational attainment** |  |  |  |
|  | Intermediate | 27 (10.3) | 1 [Reference] | **-** |
|  | College | 18 (6.9) | 0.30 (0.14-0.69) | **0.004** |
|  | Undergraduate | 120 (46.0) | 0.45 (0.25-0.81) | **0.008** |
|  | Postgraduate | 96 (36.8) | 0.79 (0.42-1.50) | 0.475 |
|  | **Living area having COVID-19 cases** |  |  |  |
|  | No | 84 (32.2) | 1 [Reference] | - |
|  | Yes | 177 (67.8) | 1.27 (0.86-1.89) | 0.228 |
|  | **Health facility levels** |  |  |  |
|  | Central health facility | 78 (29.9) | 1 [Reference] | - |
| Provincial health facility | | 91 (34.9) | 1.26 (0.77-2.06) | 0.360 |
|  | District health facility | 40 (15.3) | 1.02 (0.56-1.85) | 0.955 |
|  | Commune health center | 10 (3.8) | 1.70 (0.54-5.36) | 0.362 |
|  | Private health facility | 38 (14.6) | 0.90 (0.48-1.67) | 0.736 |
|  | Others^a^ | 4 (1.5) | 0.83 (0.23-3.06) | 0.783 |
|  | **Age groups** |  |  |  |
|  | 18-25 | 20 (7.7) | 1 [Reference] | - |
|  | 26-30 | 58 (22.2) | 1.04 (0.54-2.01) | 0.900 |
|  | 31-40 | 94 (36.0) | 1.42 (0.70-2.89) | 0.335 |
|  | >40 | 89 (34.1) | 1.77 (0.82-3.82) | 0.144 |
|  | **Working Department** |  |  |  |
|  | Community care | 7 (2.7) | 1 [Reference] | - |
|  | Treatment | 212 (81.2) | 0.79 (0.29-2.17) | 0.648 |
|  | Preventive health | 34 (13.0) | 0.61 (0.20-1.87) | 0.382 |
|  | Others^a^ | 8 (3.1) | 0.78 (0.23-2.61) | 0.683 |
|  | **Experience in being quarantined** |  |  |  |
|  | No | 218 (83.5) | 1 [Reference] | - |
|  | Yes | 43 (16.5) | 1.05 (0.64-1.72) | 0.837 |
|  | **Gender** |  |  |  |
|  | Male | 101 (38.7) | 1 [Reference] | - |
|  | Female | 160 (61.3) | 1.37 (0.99-1.91) | 0.061 |
| Note: ^a:^ Schools, universities, research institutes, pharmaceutical companies | | | | |
